# Supplementary material for: Molecular Cloning, Characterization, and Expression of a Receptor for Activated Protein Kinase C1 (RACK1) Gene in Exopalaemon carinicauda Zoea Larvae under Aroclor 1254 Stress
Source: Biology (Basel). 2024 Mar 8;13(3):174. doi: 10.3390/biology13030174 (PMC10968475; doi:10.3390/biology13030174)
Supplement: Supplementary file 1 [file biology-13-00174-s001.zip › biology-2817769-supplementary.pdf]

Supplementary Table S1. Percent identity of *Ec*RACK1 amino acid sequences compared with other species predicted by Clustal2.1 program.

Species	Similarity (%)											
Scylla paramamosain	100.00											
Exopalaemon carinicauda	**94.65**	**100.00**										
Penaeus japonicus	95.60	**96.86**	100.00									
Eriocheir sinensis	96.86	**96.54**	98.11	100.00								
Pacifastacus leniusculus	95.91	**96.86**	97.80	98.43	100.00							
Drosophila melanogaster	79.50	**79.50**	80.13	79.50	79.18	100.00						
Bombyx mori	82.65	**81.70**	82.97	82.65	82.33	87.07	100.00					
Branchiostoma belcheri	72.15	**72.78**	73.10	73.10	72.78	72.47	73.42	100.00				
Gallus gallus	77.92	**77.60**	78.86	78.55	77.92	76.66	78.23	81.33	100.00			
Homo sapiens	77.92	**77.60**	78.86	78.55	77.92	76.66	78.23	81.33	100.00	100.00		
Ruditapes philippinarum	77.92	**77.60**	78.86	78.55	77.92	76.66	78.23	81.33	100.00	100.00	100.00	
Pocillopora damicornis	74.60	**75.24**	76.19	75.56	74.92	73.33	74.29	74.92	81.90	81.90	81.90	100.00
